# Crystal Shape Factors of Form I Paracetamol

**DOI:** 10.1021/acs.cgd.5c00005

**Published:** 2025-04-18

**Authors:** Mayank Vashishtha, Juntao Li, Mahmoud Ranjbar, Srinivas Gadipelli, Paul R Shearing, Gavin Walker, K Vasanth Kumar

**Affiliations:** † Department of Chemical Sciences, Synthesis and Solid State Pharmaceutical Centre, University of Limerick, Limerick V94 T9PX, Ireland; ‡ Department of Chemical Engineering, University College London, London WC1E 6BT, United Kingdom; § Department of Engineering Science, University of Oxford, Parks Road, Oxford OX1 3PJ, United Kingdom; ∥ Department of Chemical and Process Engineering, Faculty of Engineering and Physical Sciences, University of Surrey, Guildford GU2 7XH, U.K.

## Abstract

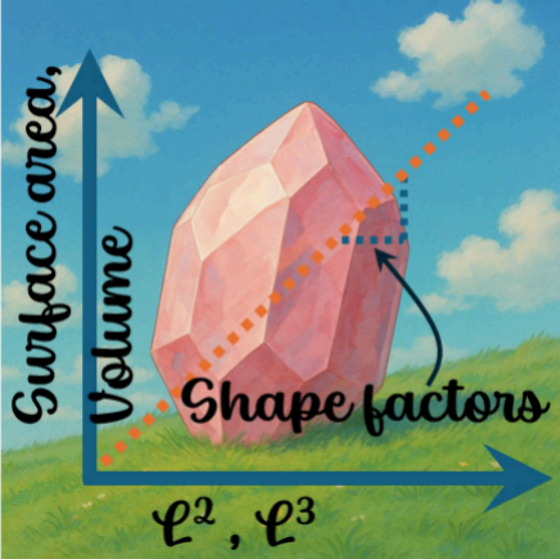

In this work, we report the new protocols that are developed to determine the shape factors of Form I paracetamol crystals using a combination of techniques that rely on the state-of-the-art X-ray computed tomography and Morphologi G3. The determined shape factors successfully predicted the crystal growth rate of paracetamol in 2-propanol and the length of the crystals growing in the supersaturated solution.

## Abstract 2

X-ray computed tomography and optical microscopy can be effectively combined to predict crystal shape factors with reasonable accuracy.

Crystal shape factors are important and are essential to accurately predict the crystal growth kinetics.^1−3^ Crystallization via crystal growth is an important operation to purify active pharmaceutical compounds.^4−7^ Crystal growth allows tailoring the crystal size and its distribution that dictates the efficacy and the operating procedures of the downstream unit operations that include filtration and drying.^8^ A typical batch crystallizer contains several millions of crystals, and it is essential to transpose the measurable quantities like the solution concentration, mass crystallized, or suspension density into the growth kinetics of crystal population.^9,10^ It is straightforward to obtain these measurable quantities using suitable analytical technology tools. However, to convert these measurable quantities to crystal growth rate, it is essential to find correlations that relate these measurable quantities with the crystal shape.^1,3^

Most organic crystals are multifaceted, with each crystal face growing at different rates during crystal growth. Theoretically, facets with smaller external surface areas grow faster than those with larger surface areas.^11−13^ When dealing with multifaceted crystals, converting measurable quantities such as concentration or mass crystallized into growth rates becomes challenging. While linear growth rates can be determined from experiments with single crystals,^14−17^ it is more practical to represent growth rates in terms of overall growth rates for the industrial crystallizers containing a large population (several thousands to millions) of crystals.^9^ Estimating linear growth rates in a population of crystals, as in industrial crystallizers, can be difficult. This is the case where the shape factors, including volume and area shape factors, become valuable. They allow for reasonably precise estimation of both linear and overall growth rates of crystals based on observed concentration or supersaturation depletion during the growth.^1,2^ Despite the importance of crystal shape factors, there are no universally established protocols for their determination, particularly for the area shape factor. Notably, the seminal work of Garside and Mullin remains the primary reference for methods to determine crystal shape factors, serving as a guideline for researchers in the field.^1^ While volume shape factors can often be estimated with relative ease, determining area shape factors remains a significant challenge due to the lack of clear and standardized methodologies. Garside and Mullin employed a labor-intensive approach, manually mapping crystal images to estimate surface areas.^1^ Given the time-consuming and labor-intensive nature of determining shape factors, researchers often adopt a simplified approach. They assume crystals are spherical or assign a definite geometry—such as rectangular prismatic, square, or rhombohedral—and apply the corresponding shape factors to predict growth rates from measurable quantities like concentration or mass crystallized.^3^ While these assumptions can provide approximate estimates of growth rates, they fail to capture the actual correlations among the mass, number, volume, and external surface area of crystals in a population.

In this work, we introduce a novel and streamlined method utilizing state-of-the-art X-ray computed tomography (XCT) to accurately determine the area shape factor. This approach is applied to paracetamol, an industrially significant compound, demonstrating its utility and potential as a model system for broader applications.

## Experimental Section

### Preparation of Good Quality Crystals

Paracetamol crystals were obtained using cooling crystallization experiments performed in batch mode. Crystallization experiments were performed using a 1000 mL reactor placed in a Mettler Toledo Optimax 1001 workstation with a reactor volume of 800 mL. All the crystal growth experiments were performed using a recipe programmed using Mettler Toledo’s iControl software. The temperature inside the crystallizer was maintained or altered by an external jacket that relies on electrical heating and solid-state cooling technology. The agitation inside the crystallizer in all the experiments was maintained at 300 rpm, and the agitation was provided using an overhead stirrer. We added 189.9 g of paracetamol to 800 mL of isopropanol. This is approximately equal to the solubility of paracetamol at 60 °C. The solution was then heated to 75 °C at a rapid cooling rate. Then we maintained the solution at this temperature for 45 min to ensure complete dissolution of the paracetamol in the solvent. Then we rapidly cooled the solution to a working temperature of −5 °C. Once the solution reached the working temperature, we maintained the reactor at the working temperature for 24 h, which is more than enough to observe a phase change via primary nucleation followed by the solution reaching saturation via the combination of nucleation and crystal growth. After 24 h, the crystallized product was separated by using a vacuum filtration setup with Whatman grade 1 qualitative filter paper. The crystals were left in the filtration unit under vacuum for at least one h within a fume hood. This crucial step minimizes the dissolution of crystal facet edges caused by residual solvent adhering to the crystals, thereby enhancing the crystal quality. After filtration, the crystals were air-dried in a fume hood for 8 h and used directly without further processing.

### Seed Crystals for the Crystal Growth Experiments

The crystal growth experiments were performed using seeds of size in the range of sieve fraction: −90+112 μm. The seeds were obtained using a sieve shaker, and the crystals were obtained from the first crystallization batch.

### Preparation and Characterization of Crystal Samples to Estimate the Volume Shape Factor

To obtain the volume shape factor, it is essential to use crystals of the same size and shape and of high-quality, specifically, defect-free, nonagglomerated and unbroken crystals. Crystals of different size fractions were obtained by using a mechanical sieve shaker (Retsch AS 200 basic). Mechanical sieving also helps to disintegrate the loosely agglomerated crystals (if any) into single crystals. From the first batch of crystals, we obtained four different sieve fractions, which includes −500+540 μm, −600+640 μm, −640–710 μm, and −710+760 μm. From the second, two sieve fractions were collected: −500+540 μm and −710+760 μm.

To determine the volume shape factor, we carefully selected good-quality crystals that were manually selected from each size fraction. We spread the crystals on the glass slide and carefully observed them under a light microscope (Olympus MLX-B Plus). We carefully selected good quality crystals of the same size (the size was measured manually using the Olympus Stream Image Analysis Software suite integrated to the microscope that allows us to measure the image in live mode while the crystals are under inspection. From each size fraction we selected from 11 to up to roughly 45 good-quality crystals of same size (shown in Table 1). We define good-quality crystals as crystals that are not agglomerated or single crystals, without any crystal breakage and defect free at the scale investigated under a light microscope. Once we managed to handpick the crystals, we saved those crystals carefully in a vial. Care was taken to avoid crystal breakage during handling for characterization pruposes. The total weight of hand-picked crystals from each fraction was then measured using a Sartorius Cubis II (MCE) Semi-Micro Analytical Balance, providing the value of *M* in eq 5 (discussed later in Linear and Overall Crystal Growth Rate) for further calculation.

**Table 1:** Number of Crystals Hand-Picked from Each Sieve Fraction, Their Total Weight, and the Mean Length Obtained Using Morphologi G3

Sieve fraction (μm)	Number of crystals	*L*_average_, cm^a^	*M*, g
–500+540	20	0.0677	2.70 × 10^–3^
–600+640	41	0.0807	9.25 × 10^–3^
–640+710	37	0.0854	1.09 × 10^–2^
–710+760	15	0.079007	3.80 × 10^–3^
–800+900	17	0.098507	7.00 × 10^–3^
–1000+1100	14	0.119576	9.40 × 10^–3^
–710+760^b^	44	0.0927	1.50 × 10^–2^
–500+540^b^	44	0.0677	5.94 × 10^–3^

a Obtained using Morphologi G3 (see Experimental Section). Note that this value is not the mean crystal size, but it corresponds to the average length of all the crystals obtained using Morphologi G3.

b Crystal samples obtained from the second crystallization batch.

### Calculating the Length of a Single Crystal or the Crystal Population

To calculate the average length of hand-picked crystals from each sieve fraction and to determine the number-based particle size distribution (PSD), a Malvern Morphologi G3 microscopic image analysis instrument was employed. Crystals were carefully spread manually on an analysis glass plate (180 × 110 mm) to avoid breakage. Any crystals showing signs of damage during handling were excluded from the analysis (example, two crystals from the sieve fraction −500+540 μm were removed due to breakage while transferring the crystals from the vial to the analysis plate). A diascopic light was passed beneath the glass plate and calibrated to an intensity setting of 80 with a tolerance of 0.20. The particle sizes of the crystals were measured using 20× and 50× magnification optics (Nikon TU Plan ELWD). Following the analysis, a standard operating procedure (SOP) was developed to define key image analysis parameters. The primary parameter, particle length, was measured as the longest projection between two points on the major axis of the particle’s two-dimensional area. The length data from the image analysis were used to construct the PSD. The average length of crystals from each sieve fraction was calculated by averaging the lengths of all crystals within the PSD for that fraction. Morphologi G3 was also used using the same recipe as that explained above to obtain the PSD of the seed crystals and the final crystals collected at the end of the crystal growth experiment.

### Preparation of Crystal Samples for Estimating the Area Shape Factor

To determine the area shape factor, crystals of varying lengths were segregated, as the surface area increases proportionally with crystal length. Crystals obtained from a wide range of sieve fractions were spread on a microscope slide. High-quality, defect-free crystals of different lengths were selected and individually stored in separate Eppendorf tubes. The length of each selected crystal was measured at the micrometer scale using Morphologi G3. These same crystals were subsequently analyzed using X-ray computed tomography (XCT) to characterize their external surface area.

### XCT

To perform this analysis, the crystals were fixed onto a plate for scanning. Images were captured by a detector and processed by a computer to generate a three-dimensional (3D) image of the internal structure of the sample. The raw data from the XCT scan were processed by the computer to reconstruct the 3-dimesional image of the internal structure of the sample. An A-Series/Compact Laser Micromachining System (Oxford Lasers, Oxford, UK) with an embedded Class 4, 532 nm wavelength laser was used to prepare samples for XCT characterization. All XCT imaging was performed by using a Zeiss Xradia 520 Versa (Carl Zeiss Microscopy Inc., Pleasanton, US) micro-CT instrument. XCT scans were carried out with an X-ray source tube voltage of 120 kV with an exposure time of 6 s per projection image for all crystals. A total of 401 projection images were collected per scan with a 4× lens for all crystals. Reconstruction of the radiographic data was achieved using a cone-beam-filtered back-projection algorithm implemented in Zeiss Scout and Scan software resulting in a reconstructed voxel size of ∼1.96 μm. Postprocessing of the reconstructed CT data was conducted using Avizo 9.4 (Thermo Fisher Scientific, UK).

### Crystal Growth Experiment

The crystal growth experiment was conducted using an EasyMax101 workstation with a reactor volume of 100 mL. Initially, 13.7865 g of paracetamol was added to 100 mL of isopropyl alcohol at room temperature. The solution was rapidly heated to 75 °C and held at this temperature for 45 min to ensure the complete dissolution of the solute. Subsequently, the solution was rapidly cooled to 30 °C. Upon reaching 30 °C, 20 wt % of seed crystals were introduced into the solution. At this temperature, the supersaturation concentration (Δ*C* = *C* – C*, where *C* is the concentration of solution at any time *t* and C* is the solubility concentration) was calculated to be 3.1815 g per 100 mL of isopropanol. The experiment was carried out under constant agitation at 250 rpm. The crystal growth process was allowed to proceed undisturbed for 24 h to ensure complete consumption of supersaturation through crystal growth. During this period, the suspension density of the solution was monitored using an in situ Raman spectroscopy tool, a part of the Process Analytical Technology suite. The Raman spectra were converted into concentration values or mass crystallized using a two-point calibration method developed and reported elsewhere.^18^

### Linear and Overall Crystal Growth Rate

The linear growth rate (*R*) and the overall growth rate (*R*_g_) of the crystal population in an industrial crystallizer can be related to the mass crystallized, volume shape factor, and number of crystals in the crystallizer and are given by the expressions^2,9,19,20^
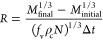
1
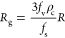
2where *f*_v_ and *f*_s_ are the volume and surface shape factors, respectively. ρ_c_ is the crystallographic density of paracetamol and is equal to 1.293 g/cm^3^. The term *f*_v_ relates the bulk volume of the growing crystals and crystal population with the measurable quantity mass crystallized as follows:
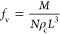
3

Equation 3 can be used to estimate the length of the crystals at any time provided we know the solid concentration in the solution at any time in the crystallizer.

The term *f*_s_ relates the increase in the external surface area of the bulk of the crystal with the population *N* using the relationship^1,2^
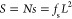
4where *M* is mass of the crystals in the crystallizer at any time *t*, *s* is the external surface area of the single crystal, and *S* is the total surface area of the crystal population in the crystallizer, ρ_c_.

The number of crystals in the crystallizer can be estimated by rewriting the expression as in eq 3
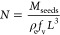
5where *M*_seeds_ in the mass of the seeds and the mean length of the seed crystals obtained from the PSD of the seeds. Note that to calculate the number of crystals in the seeds, we used the mean length of the seed crystals prepared using recrystallization experiments discussed above, whereas to estimate the surface and volume shape factors we used the average length of all the hand-picked crystals in each size fraction.

## Results and Discussion

Within the context of crystallization, the calculation of linear growth rates requires the volume shape factor, while determining the overall growth rate of the crystal population necessitates both the area and volume shape factors (refer to the Experimental Section for details). Briefly, the volume shape factor *f*_*v*_ can be correlated to the mass of the crystals in *M*, crystal population *N*, density of the crystals ρ, and the length of the crystal *L* as shown in eq 3, whereas the area shape factor *f*_s_ can be correlated to the length and the external surface area of the bulk crystal (see eq 4). Mathematically, eq 3 enables the estimation of the volume shape factor, provided the relationships among the mass of crystals (*M*), the number of crystals in the population (*N*), the crystallographic density (ρ), and the average length of the crystals (*L*) are known. Similarly, eq 4 allows for the estimation of the area shape factor given the length of each crystal and its corresponding external surface area.

The relationship between *M*, *N*, ρ, and *L* and the volume shape factor was determined using an advanced optical microscopy technique (see Experimental Section), while the quantitative relationship between the surface area (*s*) and *L* was obtained for the first time using a state-of-the-art X-ray computed tomography technique. To calculate the volume and area shape factors, it is crucial to prepare high-quality crystals, ideally flawless at the microscopic scale. To determine the volume shape factor, crystals of different size fractions must be selected, and within each size fraction, the crystals should exhibit narrow size distributions. This ensures the relationship between mass, length, number, and density of the crystals is accurately captured by the volume shape factor. To achieve this, we developed a meticulous experimental protocol involving batch recrystallization of as-received paracetamol followed by mechanical sieving to obtain crystals of distinct size fractions (details in the Experimental Section). Six size fractions were selected: −500+540 μm, −600+640 μm, −640+710 μm, −710+760 μm, −800+900 μm, and −1000+1100 μm. Additionally, two size fractions (−500 + 540 μm and −710 + 760 μm) were characterized from a second crystallization batch.

**Figure 1:**
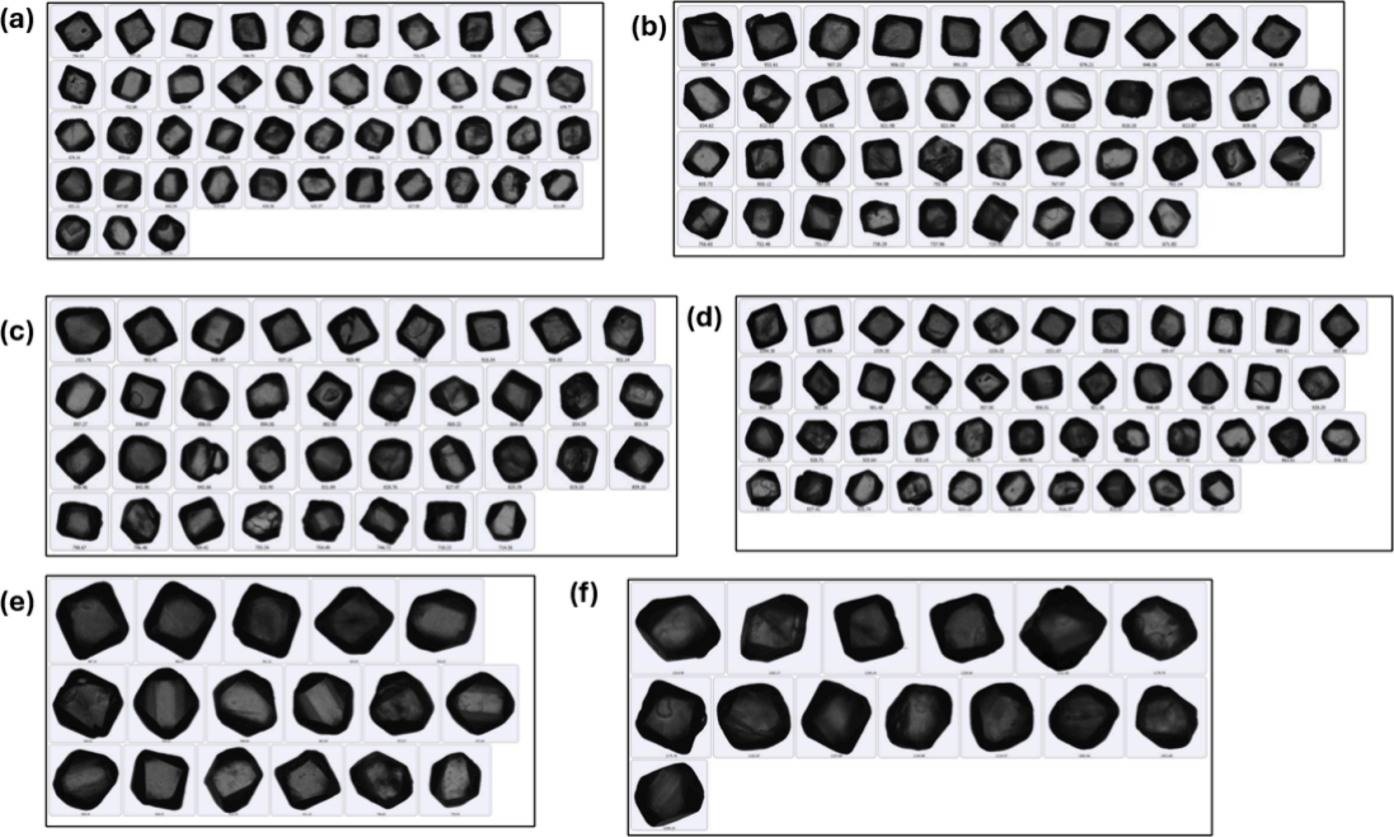
Microscopic images of all the handpicked crystals from different sieve fractions: (a) −500+540 μm, (b) −600+640 μm, (c) −640+710 μm, (d) −710+760 μm, (e) −800+900 μm, (f) −1000+1100 μm. (a–f) crystals were obtained from the first recrystallization batch.

From each sieve fraction, between 14 and 44 crystals were carefully handpicked under an optical microscope, depending on the crystal size. Crystals from a second batch of recrystallization were also sieved into size fractions, and the handpicking process was repeated. Strict care was taken to ensure that all hand-picked crystals were visually defect-free under the microscope. Although this process is labor-intensive, it is critical for obtaining high-quality experimental data, as it ensures that all selected crystals within each size fraction are flawless and nearly identical in length. The handpicked crystals from each size fraction across both recrystallization batches were weighed to determine their total mass. These crystals were then characterized for their morphology and key morphological properties, primarily focusing on their length. Microscopic images of the handpicked crystals from selected sieve fractions, obtained using Morphologi G3, are presented in Figure 1a–f (see Experimental Section). The images clearly show that the handpicked crystals are of excellent quality and are free from visible defects and agglomeration. More importantly, the uniformity in size within each fraction is evident, which is crucial for obtaining accurate and precise values of the volume shape factor.

**Figure 2:**
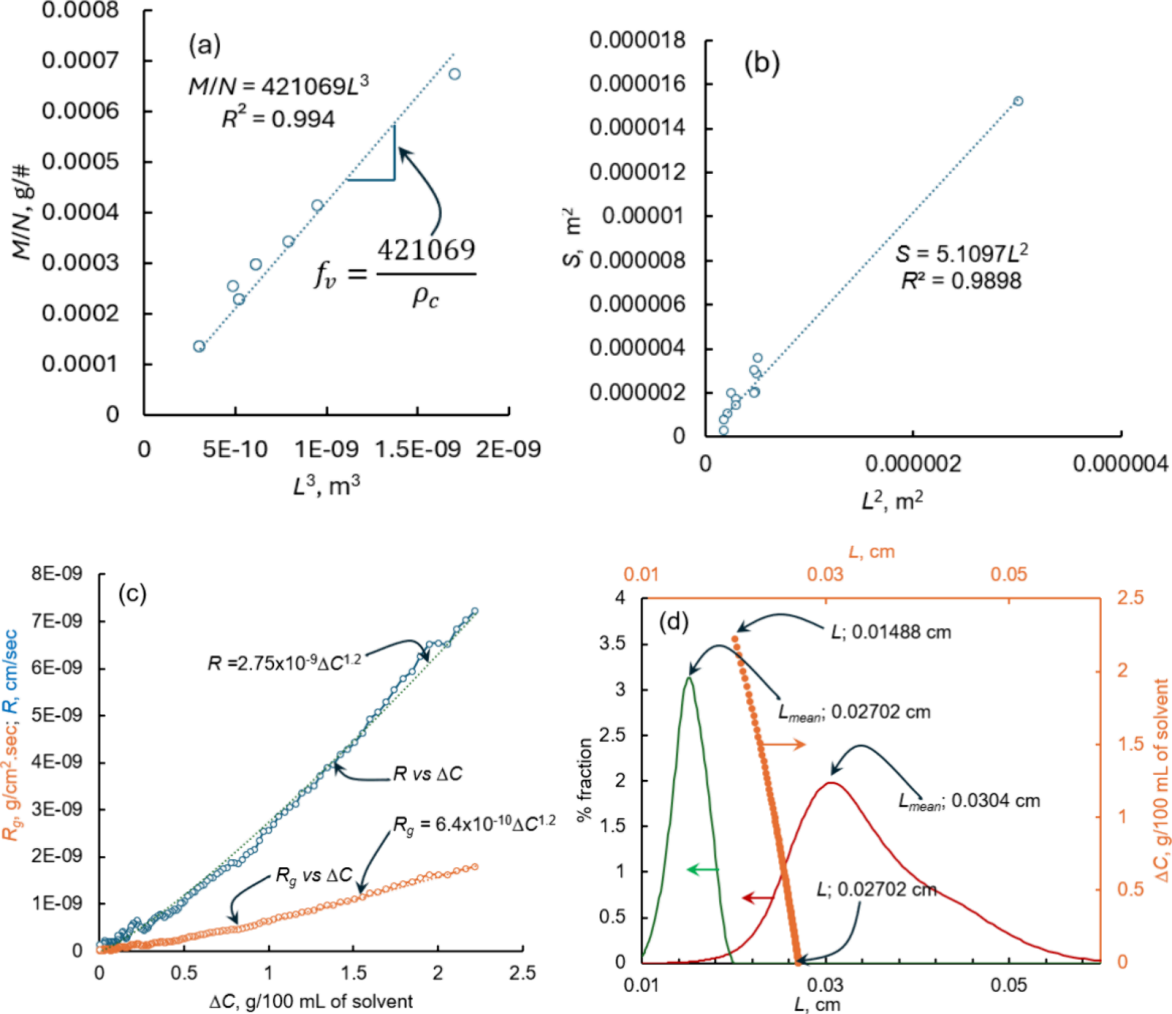
(a) Plot of *M*/*N* versus *L*^3^ (note: *L* obtained from Morphologi G3). (b) Plot of *S* obtained from XCT versus *L*^2^ obtained using Morphologi G3. (c) Plot of the linear and overall growth rate versus supersaturation (Δ*C* = *C* – C^*^). (d) Plot of % fraction versus length (bottom-left axis) of the crystals measured using Morphologi G3 and plot of the length of the final crystals (obtained using shape factors and mass balance versus supersaturation (top-right axis); (green —, PSD of the seed crystals; red —, PSD of the final crystals collected from the crystal growth experiment; orange ●, mean length of crystals growing in the supersaturated solution).

In Table 1, we provide the number of crystals handpicked from each size fraction obtained from two crystallization batches, their average length, and the corresponding weight of those crystal collective under each size fraction. Using these data, we obtained a statistically precise value for the volume shape factor from the plot of *M*/*N* versus *L*^3^ using eq 3 as shown in Figure 2a. The plot of *M*/*N* versus *L*^3^ exhibits perfect linearity with the intercept passing through the origin. The slope of this plot, according to eq 3, provides the volume shape factor. For paracetamol crystals, as shown in Figure 2a, the volume shape factor was determined to be 0.3264. This volume shape factor enables the estimation of the total number of paracetamol crystals in a population, provided that the mass of the crystals and their mean length are known.

To calculate the area shape factor, we propose to use a state-of-the-art XCT technique. To calculate the area shape factor, we carefully hand-picked 12 crystals from the product obtained from the first crystallization batch (Experimental Section). The hand-picked crystals were characterized for their morphology and for their length using Morphologi G3 (Experimental Section). The lengths of the crystals obtained using Morphologi G3 are given in Table 2. Once the length of the crystals was obtained, we characterized these crystals for their external surface area using XCT (Experimental Section). The external surface area, the images obtained using XCT, and the length of these crystals measured using Morphologi G3 are given in Table 2. The microscopic images and the images obtained using XCT of the crystals selected to characterize the surface shape factor are also given in Table 2. From Table 2, it is evident that all the crystals selected for the study are defect free, which make them suitable candidates for the characterization of the surface shape factor.

**Table 2:**
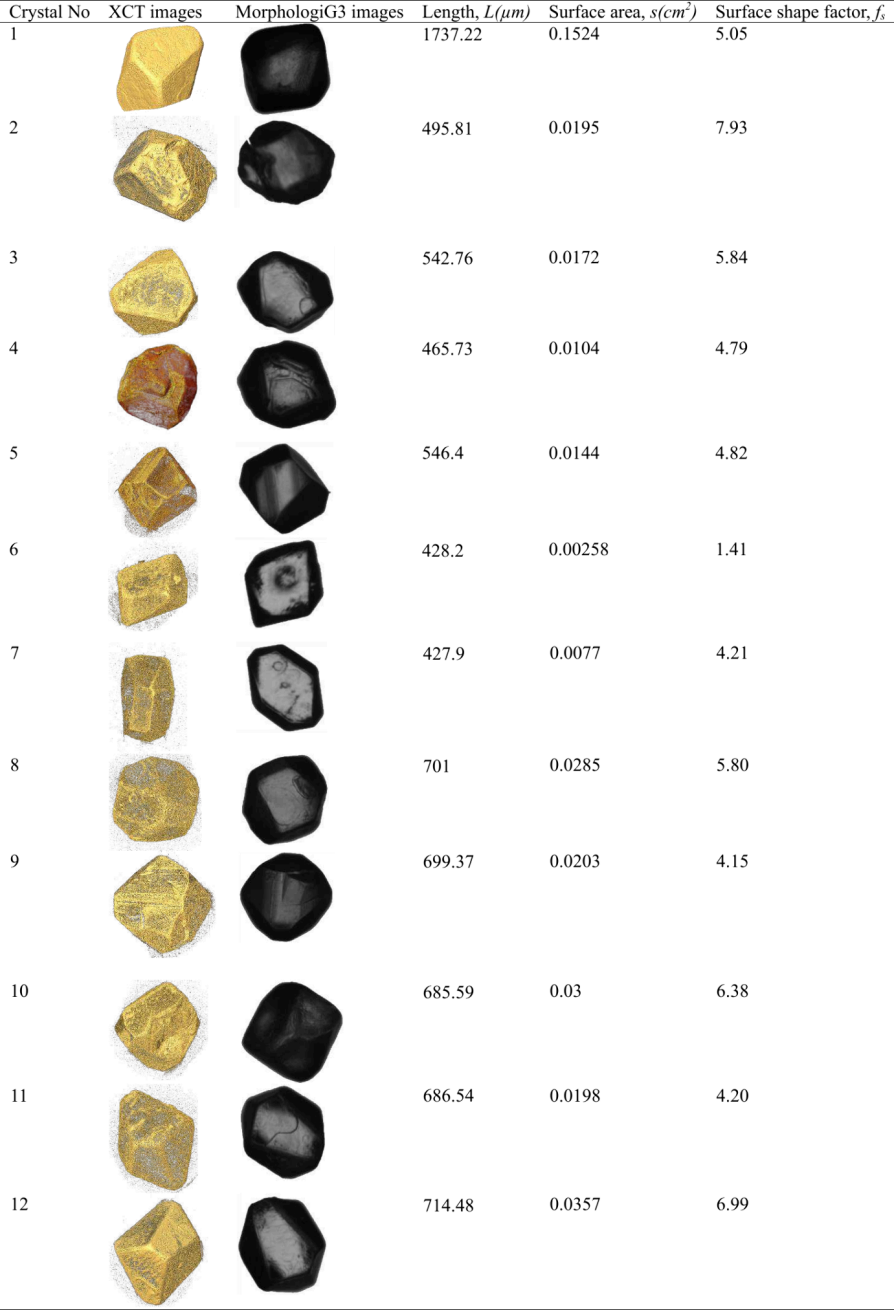
Images of the Crystals Obtained Using morphologiG3 and XCT, Length of the Crystals Obtained Using Morphologi G3, and the Surface Shape Factor of Each Crystal

To calculate the area shape factor, we plotted the external surface area obtained from XCT versus the length raised to the power of two of the crystals obtained using Morphologi G3. From Figure 2b, it is clear that there exists a linear relationship between *S* versus *L*^2^ and the trendline cuts through the origin. The surface shape factor was then estimated from the slope of this plot using eq 4 and was found to be equal to 5.1097. This value is useful as it allows calculating the external surface area of the paracetamol crystals if we know the length of the crystal, which can be easily obtained using Morphologi G3 or a simple light microscope.

It is worth noting that crystals are anisotropic in nature, with each facet growing at a different rate depending on its attachment energy, which ultimately determines the crystal’s overall morphology. Typically, the fastest-growing facet develops the smallest surface area, resulting in shape anisotropy. This morphology should be precisely quantified using shape factors that can accurately predict the crystal’s actual length, external surface area, and volume, based on measurable parameters like concentration or suspension density. In the absence of accurate shape factors, crystals are often approximated as spheres with corresponding shape factors applied. However, this assumption is unrealistic for most pharmaceutical crystals, such as paracetamol, which are multifaceted. Therefore, we believe that the shape factors obtained in this study provide a more accurate method for calculating the length, surface area, and volume of the crystals. It should be noted that if the shape factors are accurately estimated, they can even be used to quantitatively estimate solution concentration or suspension density using a simple mass balance, provided we have information about the length of the crystals in the population which can be obtained using offline analysis of crystals or even using sophisticated process analytical technology tools like Particle Visual Measurement systems. As XCT provided precise 3D reconstructions of individual crystals, we believe that the shape factors derived from this technique are statistically robust, enabling direct computation of volume, external surface area, and crystal growth kinetics, capabilities that are not achievable when using shape factors that correspond to the spherical particles.

To show the usefulness of the predicted shape factors, we calculated the overall growth rates of paracetamol growing in a supersaturated solution with supersaturation, *S* = *C*/C^*^ = 1.3 or Δ*C* = *C* – C^*^ = 13.7865 – 10.605 = 3.1815 g/100 mL of isopropanol, starting from an average seed size of 1.4885 × 10^2^ cm. In Figure 2c we show the plot of the linear growth rate versus supersaturation (see the Experimental Section) and the overall growth rate versus the supersaturation (both calculated using the shape factors obtained above). It is clear that both the linear growth rate and the overall growth rate increases with the increase in the supersaturation. This can be expected, considering the fact that the crystal growth rate is driven by supersaturation and proportional to the driving force, in this case supersaturation. The relationship between the growth rate and the supersaturation follows a power law type of expression. This agrees with the crystal growth versus supersaturation relationship reported for several organic and inorganic compounds. The magnitude of the linear growth rate ranges from 0 to 10^–9^ g/(cm^2^ s) at the studied range of supersaturation shown in Figure 2c. The overall growth rate ranges from 0 to 10^–8^ cm/s (Figure 2c). Clearly the determined shape factors are useful to predict the linear growth rate (which requires a volume shape factor) and overall growth (which requires both a volume and area shape factor) rate. To complement the results shown in Figure 2c which are obtained based on the shape factors calculated in this study, we also calculated the length of the final crystals using the obtained shape factor. In Figure 2d we showed the particle size distribution (PSD) of the seeds and the final crystals collected once the solution reached the solubility concentration via crystal growth. Furthermore, in Figure 2d we also show the length of the crystals versus the supersaturation in the solution. The PSD clearly shows that the size distribution remains unimodal before and after the crystal growth, which indicates no nucleation, and the consumption of the supersaturation is only due to the crystal growth. The PSD of the final crystals exhibits a peak at 3.05 × 10^–2^ cm which can be taken as the mean size of the final crystals which is almost close to the length of the final crystals calculated using the shape factors (was found to be equal to 2.7023 × 10^–2^ cm) obtained in this study. The difference between the mean crystal size obtained using the microscopic technique and those predicted using the shape factor was found to be only around 11%. Clearly the shape factors accurately predicted the length of the final crystals, which means the determined volume shape factor should successfully predict the linear growth rate with a good level of accuracy.

## Conclusions

To conclude, we estimated the shape factors for the industrially significant compound and crystallization workhorse material, paracetamol. For the first time, we introduced a method to calculate the surface area shape factor using XCT. These shape factors were effectively applied to determine the overall growth rate and linear growth rate of paracetamol in isopropanol. The results and protocols presented here provide a valuable framework for characterizing crystalline materials in terms of their area and volume shape factors. Notably, the mean lengths of the final crystals obtained from batch crystal growth experiments, calculated using the shape factors determined in this study, closely matched the mean length measured through microscopic techniques. The difference between these two methods was approximately 11%, underscoring the reliability and accuracy of the proposed approach.

The protocols developed in this study require high-quality, well-formed crystals and careful sample preparation. This should not be viewed as a limitation but rather as a reflection of standard industrial practice, where seed crystals with uniform morphology are routinely generated through controlled nucleation and sieving. Crystal growth experiments are typically performed within the Metastable Zone Width (MSZW) and near solubility conditions under ideal mixing, minimizing nucleation, breakage, and agglomeration. Under such conditions, the use of defect-free crystals is both expected and necessary to ensure accurate kinetic modeling and reliable process performance. Our approach, though demonstrated using paracetamol, is generalizable across different APIs and solvents, provided crystal growth is the dominant mechanism. While Feret diameter and other two-dimensional morphological parameters can be obtained using standard microscopy techniques (similar to Morphologi G3), accurate estimation of surface area shape factors necessitates advanced imaging such as XCT. This work is the first to demonstrate the applicability of XCT for shape factor determination in crystallization studies. Although the current study utilized a limited number of crystals for surface area estimation, the statistical robustness can be improved by analyzing larger crystal populations. Overall, the methodology and instrumentation proposed here provide a universal and scalable framework for shape factor determination, essential for robust modeling of crystal growth kinetics and process control in pharmaceutical manufacturing.

## References

[ref1] Garside J.; Mullin J. W.; Das S. N. Importance of Crystal Shape in Crystal Growth Rate Determinations. Ind. &Amp; Eng. Chem. Process Des. Dev. 1973, 12 (3), 369–371. 10.1021/i260047a027.

[ref2] Crystallization, 4th ed.; Mullin J. W., Ed.; Butterworth-Heinemann: 2001.

[ref3] Garside J.; Mersmann A.; Nývlt J. Measurement of Crystal Growth and Nucleation Rates; Institution of Chemical Engineers (IChemE): 2002.

[ref4] Vasanth Kumar K. Crystallisation fundamentals; nucleation & growth. https://www.youtube.com/watch?v=VhDE9xSmahg&list=PL0B26A0FGHaMkX_8XKSHN7qatec5DhciT.

[ref5] Vashistha M.; Cliffe C.; Murphy E.; Palanisamy P.; Stewart A.; Gadipelli S.; Howard C. A.; Brett D. J. L.; Kumar K. V. Dotted Crystallisation: Nucleation Accelerated, Regulated, and Guided by Carbon Dots. CrystEngComm 2023, 25 (33), 4729–4744. 10.1039/D3CE00574G.

[ref6] Vashishtha M.; Kakkar S.; Ranjbar M.; Kumar K. V. Crystallisation: Solving Crystal Nucleation Problem in the Chemical Engineering Classroom Based on the Research Grade Experiments Deployed in Virtual Mode. Educ. Chem. Eng. 2024, 49, 12–25. 10.1016/j.ece.2024.07.001.

[ref7] Chen J.; Sarma B.; Evans J. M. B.; Myerson A. S. Pharmaceutical Crystallization. Cryst. Growth Des. 2011, 11 (4), 887–895. 10.1021/cg101556s.

[ref8] Wood B.; Girard K. P.; Polster C. S.; Croker D. M. Progress to Date in the Design and Operation of Continuous Crystallization Processes for Pharmaceutical Applications. Org. Process Res. Dev. 2019, 23 (2), 122–144. 10.1021/acs.oprd.8b00319.

[ref9] Kannuchamy V. K. Transfer of impurities into crystals in industrial processes, PhD Thesis, University of Porto, 2010.

[ref10] Mersmann A. Crystallization Technology Handbook, 1st ed..; Mersmann A., Ed.; CRC Press: 2001. 10.1201/9780203908280.

[ref11] Zumstein R. C.; Rousseau R. W. Growth Rate Dispersion in Batch Crystallization with Transient Conditions. AIChE J. 1987, 33, 1921 10.1002/aic.690331123.

[ref12] Shiau L. -D; Berglund K. A. Growth Rate Dispersion in Batch Crystallization. AIChE J. 1990, 36, 1669 10.1002/aic.690361107.

[ref13] Bohlin M.; Rasmuson Å. C. Modeling of Growth Rate Dispersion in Batch Cooling Crystallization. AIChE J. 1992, 38, 1853 10.1002/aic.690381202.

[ref14] Ottoboni S.; Chrubasik M.; Bruce L. M.; Nguyen T. T. H.; Robertson M.; Johnston B.; Oswald I. D. H.; Florence A.; Price C. Impact of Paracetamol Impurities on Face Properties: Investigating the Surface of Single Crystals Using TOF-SIMS. Cryst. Growth Des. 2018, 18 (5), 2750–2758. 10.1021/acs.cgd.7b01411.

[ref15] Yang Y.; Xu S.; Xie M.; He Y.; Huang G.; Yang Y. Growth Mechanisms for Spherical Mixed Hydroxide Agglomerates Prepared by Co-Precipitation Method: A Case of Ni1/3Co1/3Mn1/3(OH)2. J. Alloys Compd. 2015, 619, 846–853. 10.1016/j.jallcom.2014.08.152.

[ref16] Ristic R I; Sherwood J N The Growth Rate Variation of the (100) Faces of Adp Crystals in the Presence of Manganese Ions. J. Phys. D. Appl. Phys. 1991, 24 (2), 171–175. 10.1088/0022-3727/24/2/013.

[ref17] Guzman L. A.; Maeda K.; Hirota S.; Yokota M.; Kubota N. Unsteady-State Impurity Effect of Chromium (III) on the Growth Rate of Potassium Sulfate Crystal in Aqueous Solution. J. Cryst. Growth 1997, 181 (3), 272–280. 10.1016/S0022-0248(97)00161-9.

[ref18] Kumar K. V.; Ramisetty K. A.; Devi K. R.; Krishna G. R.; Heffernan C.; Stewart A. A.; Guo J.; Gadipelli S.; Brett D. J. L.; Favvas E. P.; Rasmuson Å. C. Pure Curcumin Spherulites from Impure Solutions via Nonclassical Crystallization. ACS Omega 2021, 6, 23884 10.1021/acsomega.1c02794. 34568668 PMC8459370

[ref19] Vasanth Kumar K.; Gadipelli S.; Ramisetty K. A.; Heffernan C.; Stewart A. A.; Ranade V.; Howard C.; Brett D. Nonclassical Crystal Growth and Growth Rate Hysteresis Observed during the Growth of Curcumin in Impure Solutions. CrystEngComm 2023, 25 (23), 3361–3379. 10.1039/D3CE00051F.

[ref20] Ranjbar M.; Vashishtha M.; Gadipelli S.; Ramisetty K.; Walker G.; Brett D. J. L.; Kumar K. V. Pseudo Second Order Kinetic Expression to Predict the Kinetics of the Anomalous Crystal Growth of Curcumin in Impure Solution. CrystEngComm 2024, 26 (8), 1077–1089. 10.1039/D3CE00624G.

